# Fulminant myocarditis associated with severe dengue in a young patient from the Peruvian Amazon: case report and literature review

**DOI:** 10.17843/rpmesp.2025.424.14881

**Published:** 2025-12-03

**Authors:** Edgar A. Ramirez-García, María José V. Canchanya-Olimar, Gian F. Montenegro-Matute, Eliana E. Varela-Meza, Vicente Mesones-Ramirez, Christian Paucar-Gonzales, Dayanna Reyes-Correa, Alex G. Ramirez-Fernández, Juan C. Celis-Salinas, Martín Casapía-Morales

**Affiliations:** 1 Universidad Nacional de la Amazonía Peruana, Iquitos, Peru. Universidad Nacional de la Amazonía Peruana Universidad Nacional de la Amazonía Peruana Iquitos Peru; 2 Departamento de Enfermedades Infecciosas y Tropicales del Hospital Regional de Loreto; Iquitos, Peru. Departamento de Enfermedades Infecciosas y Tropicales Hospital Regional de Loreto Iquitos Perú

**Keywords:** Dengue, Heart, Cardiac Arrhythmias, Severe Dengue

## Abstract

Dengue is an endemic viral infection of tropical regions that can present with clinical presentations ranging from mild forms to severe complications. Dengue-associated myocarditis, although uncommon, is a potentially fatal complication that should be suspected in febrile patients from endemic areas presenting with signs of cardiovascular dysfunction. We present the case of a 21-year-old male, resident in the Peruvian Amazon, who developed acute heart failure and refractory cardiogenic shock secondary to dengue myocarditis, with a fatal outcome on the third day of hospitalization. This case highlights the importance of early recognition of myocardial involvement and timely cardiovascular monitoring to reduce mortality associated with this infection.

## INTRODUCTION

Dengue is an endemic disease in more than 100 countries, with nearly 50% of the world’s population at risk. In the last five decades, its incidence has increased approximately 30 times, making it a global public health problem. In the Americas, Argentina and Brazil concentrate the highest burden of cases, and according to the Pan American Health Organization, more than 5.2 million cases and about 1800 deaths were reported during 2024 ^1^.

The proposed pathophysiological mechanisms for dengue-associated myocarditis include direct viral invasion of the myocardium, exacerbated systemic inflammatory response, and hypoperfusion secondary to shock due to capillary leakage. Despite these findings, the association of myocarditis and dengue constitutes an uncommon manifestation, reported in only 3 - 5% of severe cases ^2^. Documented reports in Latin America, especially in tropical regions like the Peruvian Amazon, remain scarce, which highlights the importance of communicating the present case.

Dengue is caused by four serotypes of the dengue virus (DENV-1, DENV-2, DENV-3 and DENV-4), of the genus Flavivirus. It is primarily transmitted by the bite of Aedes mosquitoes, which breed in stagnant water and are more active during the day ^3^. This disease constitutes a major global public health problem, with an estimated burden of more than 390 million annual infections, according to recent epidemiological models ^4^. The infection presents a clinical spectrum that varies from asymptomatic forms to severe clinical presentations with a fatal outcome ^3^^,^^5^. The World Health Organization classifies the disease into: dengue without warning signs (fever, headache, retro-orbital pain, myalgias, arthralgias, nausea, vomiting, and rash), dengue with warning signs (severe abdominal pain, persistent vomiting, fluid accumulation, bleeding, lethargy, or hepatomegaly), and severe dengue, characterized by plasma extravasation, severe hemorrhages, or significant organ damage ^5^^,^^6^.

Although dengue is mainly associated with fever and hemorrhagic manifestations, it can compromise the cardiovascular system, causing myocarditis, arrhythmias, pericarditis, and ventricular dysfunction, with a variable incidence from 0.3% to 71% ^7^.

Myocarditis is an uncommon but potentially serious complication, which manifests with dyspnea, fatigue, and chest pain. Its diagnosis is based on clinical findings, electrocardiogram, echocardiography, and cardiac biomarkers such as troponin ^8^. Arrhythmias have also been reported, including sinus bradycardia, supraventricular tachycardia, and atrioventricular blocks, which are usually transient ^9^. Furthermore, ventricular dysfunction can manifest with a reduction in the ejection fraction, detected by echocardiography ^10^.

The proposed pathophysiological mechanisms include direct viral invasion of the myocardium, an exacerbated immune response, and hemodynamic alterations secondary to hypovolemic shock ^11^. Treatment is supportive, with hemodynamic monitoring and careful fluid management. In severe cases, specific treatment for arrhythmias or mechanical support may be required. Most cardiovascular complications are reversible after the resolution of the infection ^12^.

Our report aims to present a case of dengue-associated myocarditis in a young patient in an endemic region, describing its clinical presentation, evolution, and outcome, with the purpose of highlighting the need for early diagnosis and timely management of cardiovascular complications in the context of dengue.

## CLINICAL CASE

### Patient information

**Figure 1:**
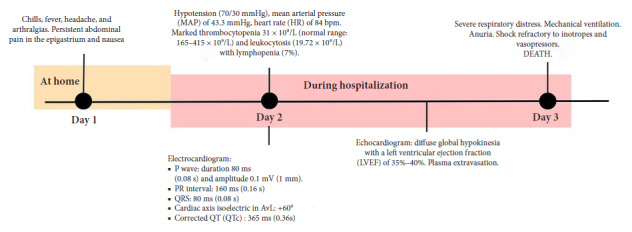
Timeline of the clinical course of the case

A 21-year-old male, native and resident of Iquitos (Peru), with no history of pathological, surgical conditions, or consumption of alcohol, tobacco, or drugs. He did not report recent travel or relevant occupational exposure. He consulted for a sudden clinical presentation of one day of evolution characterized by fever, chills, headache, arthralgias, nausea, and persistent abdominal pain in the epigastrium. Upon admission, he was observed to be in poor general condition, with toxic facies, lethargy, and cold extremities (figure 1).

### Clinical findings

**Figure 2:**
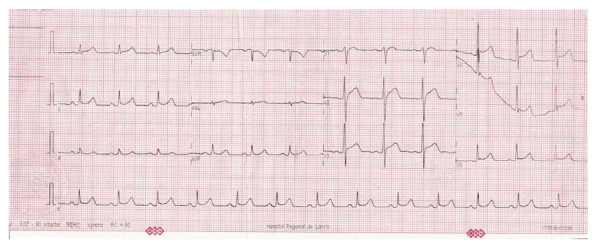
Electrocardiogram: sinus rhythm and heart rate: 78 beats/min. P wave: duration 80 ms (0.08 s) and amplitude 0.1 mV (1 mm). PR interval: 160 ms (0.16s). QRS: 80ms (0.08s). Cardiac axis: isoelectric in aVL: +60°. Corrected QT (QTc): 365ms (0.36).Abnormal findings included PR segment depression predominantly in leads I and II, diffuse ST-segment elevation of 0.5 mm in limb leads, and greater than 1 mm in precordial leads.

Initial vital signs showed: blood pressure (BP) 70/30 mmHg, mean arterial pressure (MAP) 43.3 mmHg, heart rate (HR) 84/min, respiratory rate (RR) 22/min, temperature 36.5 °C, and oxygen saturation (SatO2) of 99%. Physical examination showed pale and cold skin in distal extremities, as well as pain upon deep palpation in the epigastrium, without signs of peritoneal irritation. Initial laboratory tests reported: platelets: 31 × 10⁹/L; leukocytes: 19.72 × 10⁹/L with lymphopenia (7%); AST: 608 U/L; ALT: 381 U/L. Rapid serology showed negative IgM, positive IgG, and negative NS1 antigen for dengue. The electrocardiogram showed sinus rhythm (HR: 78/min), PR segment depression in DI and DII, along with diffuse ST-segment elevation (0.5 mm in limb leads and >1 mm in precordial leads). QRS measured 80 ms, the axis was approximately +60°and QTc was 365 ms (Figure 2). The chest X-ray showed cardiomegaly (cardiothoracic index: 0.56) and pulmonary vascular redistribution.

### Diagnostic evaluation

Given the presence of hypotension, abdominal pain, and severe thrombocytopenia, dengue with warning signs was initially considered. The electrocardiographic findings (PR segment depression and diffuse ST elevation), along with the systolic dysfunction subsequently observed, were compatible with probable acute myocarditis.

During the stay in the intensive care unit (ICU), an echocardiogram was performed which showed diffuse global hypokinesia and left ventricular ejection fraction (LVEF) of 35-40%. Cardiac biomarkers showed CPK-MB of 72.3 ng/mL (elevated). Polymerase chain reaction (PCR) for dengue was positive for DENV-2 the next day, confirming the viral etiology. Endomyocardial biopsy was not available.

### Therapeutic intervention

Upon admission, intravenous hydration was started according to the protocol for dengue with warning signs, with transient improvement in blood pressure. Two hours later, blood pressure dropped to 80/50 mmHg, leading to transfer to the ICU.

In the ICU, support with vasopressors and inotropes was instituted due to persistent hypotension. Subsequently, the patient developed severe respiratory distress, requiring mechanical ventilation. Advanced supportive management for mixed shock (distributive and cardiogenic) associated with myocarditis was initiated.

### Follow-up and outcome

Despite vasoactive support at maximum doses, the patient progressed to anuria and refractory shock. Hemodynamic deterioration progressed rapidly, and the patient died a few hours after the diagnosis of myocarditis associated with DENV-2 infection had been established.

### Patient perspective

It was not possible to gather the patient’s perspective due to his fatal outcome; however, confidentiality is respected, and the publication is justified by its scientific and educational value.

## DISCUSSION

Dengue, a viral disease transmitted by the Aedes aegypti mosquito, mainly affects tropical and subtropical regions. Although most cases are mild, some may develop severe complications, such as cardiovascular alterations, which are uncommon but concerning ^12^. Myocarditis, a fulminant complication of severe dengue, can compromise both the structure and function of the heart, causing heart failure or arrhythmias. This phenomenon is associated with increased morbidity, especially in patients previously infected with other serotypes of the virus ^12^.

Cardiac manifestations associated with dengue constitute an uncommon complication; however, they must be considered, since plasma leakage can severely compromise the cardiovascular system and lead to fatal outcomes ^13^. In the present case, the findings of global hypokinesia and ventricular dysfunction suggest myocardial damage, which is consistent with previous reports. A similar case described in a 49-year-old Asian patient presented acute fulminant myocarditis with initial involvement of the right ventricle and progression to the left ventricle, culminating in cardiogenic shock and death ^13^. Furthermore, an epidemiological study in Cuba identified electrocardiographic alterations in a significant percentage of patients with dengue, highlighting rhythm disorders in 16.9% of the cases studied ^14^^,^^15^.

As indicated, dengue can cause functional deterioration of the myocardium, both in the acute phase and in more severe forms of the disease. In this patient, the identification of diffuse global hypokinesia and a reduced left ventricular ejection fraction through echocardiography suggests the possible presence of myocarditis. This aligns with the literature, which documents that left ventricular (LV) dysfunction can be caused by subclinical myocarditis, myocardial edema, or circulatory mechanisms affecting myocardial contractility ^11^.

The cases show rare cardiovascular manifestations in severe dengue, such as heart failure and arrhythmias. The findings of hypokinesia and electrocardiographic alterations coincide with what is described in the literature, where it is underlined that these complications are usually underestimated and require close clinical surveillance. In particular, regional reviews have highlighted the importance of adequate monitoring in patients with severe dengue due to the risk of progression to significant cardiac dysfunction ^16^.

It is important to mention that the patient had an unfavorable outcome and shared electrocardiographic abnormalities with another case reported in a young male in Argentina, who developed fulminant dengue myocarditis, with an echocardiographic pattern compatible with Brugada type 1 and a fatal outcome. This finding reinforces the evidence that males might be more susceptible to developing complications during the course of the disease, compared to females ^17^.

### Implications for public health

In Loreto, up to Epidemiological Week 10 of 2025, more than 4100 cases of dengue were reported, with a predominance of forms without warning signs ^18^, but with a sustained increase in cases with warning signs and severe dengue, in a context classified as an epidemic zone. This situation demonstrates the need to strengthen clinical surveillance and the early identification of warning signs, since their timely recognition allows for the prevention of serious complications such as myocarditis and the reduction of associated mortality ^19^. The presented case demonstrates how the lack of early detection of cardiac involvement can influence fatal outcomes, underlining the urgency of optimizing the clinical management of dengue in hospitals in the Peruvian Amazon.

Among the main limitations of this report is the lack of tissue confirmation, due to the impossibility of performing an autopsy. The absence of a postmortem study prevented the confirmation of the histopathological diagnosis and the exclusion of differential diagnoses. Likewise, it was not possible to include the patient’s perspective, a fact already consigned previously in the manuscript, due to his fatal outcome.

In conclusion, the need to include electrocardiograms and echocardiograms in the clinical evaluation of patients with dengue is highlighted, as the early detection of cardiac alterations can improve the prognosis. Given that this disease is endemic in tropical areas, it is crucial that health professionals actively monitor cardiovascular complications and deepen their research, with the aim of comprehensively addressing both the viral infection and its extrapulmonary manifestations, particularly the cardiac ones, to reduce the associated morbidity and mortality.
