# Supplementary material for: Fulminant myocarditis associated with severe dengue in a young patient from the Peruvian Amazon: case report and literature review
Source: Rev Peru Med Exp Salud Publica. 2025 Dec 3;42(4):442–6. doi: 10.17843/rpmesp.2025.424.14881 (PMC12879975; doi:10.17843/rpmesp.2025.424.14881)
Supplement: Translation

# Translation: Miocarditis fulminante asociada a dengue grave en un paciente joven de la Amazonía peruana: reporte de caso y revisión de la literatura

**Authors:** Edgar A. Ramirez-García, María José V. Canchanya-Olimar, Gian F. Montenegro-Matute, Eliana E. Varela-Meza, Vicente Mesones-Ramirez, Christian Paucar-Gonzales, Dayanna Reyes-Correa, Alex G. Ramirez-Fernández, Juan C. Celis-Salinas, Martín Casapía-Morales

**Affiliations:** 1 Universidad Nacional de la Amazonía Peruana, Iquitos, Perú.; 2 Department of Infectious and Tropical Diseases of the Hospital Regional de Loreto; Iquitos, Perú.

**Keywords:** Dengue, Corazón, Arritmias Cardíacas, Dengue Grave

## Abstract

El dengue es una infección viral endémica de regiones tropicales que puede presentarse con manifestaciones clínicas que van desde formas leves hasta complicaciones severas. La miocarditis asociada al dengue, aunque poco frecuente, constituye una complicación potencialmente fatal que debe sospecharse ante la presencia de signos de disfunción cardiovascular en pacientes febriles de zonas endémicas. Se presenta el caso de un varón de 21 años, residente en la Amazonía peruana, que desarrolló insuficiencia cardíaca aguda y shock cardiogénico refractario secundario a miocarditis por dengue, con desenlace fatal al tercer día de hospitalización. Este caso resalta la importancia del reconocimiento temprano de la afectación miocárdica y del monitoreo cardiovascular oportuno para reducir la mortalidad asociada a esta infección.

## INTRODUCCIÓN

El dengue es una enfermedad endémica en más de 100 países, con cerca del 50% de la población mundial en riesgo. En las últimas cinco décadas, su incidencia se ha incrementado aproximadamente 30 veces, convirtiéndose en un problema global de salud pública. En América, Argentina y Brasil concentran la mayor carga de casos, y según la Organización Panamericana de la Salud, durante el 2024 se notificaron más de 5,2 millones de casos y cerca de 1800 muertes ^1^.

Entre los mecanismos fisiopatológicos propuestos para la miocarditis asociada al dengue se incluyen la invasión viral directa del miocardio, la respuesta inflamatoria sistémica exacerbada y la hipoperfusión secundaria al shock por fuga capilar. A pesar de estos hallazgos, la asociación de miocarditis y dengue constituye una manifestación infrecuente, reportada en solo el 3 - 5% de casos graves ^(^^2^. Los informes documentados en Latinoamérica, especialmente en regiones tropicales como la Amazonía peruana, siguen siendo escasos, lo que resalta la importancia de comunicar el presente caso.

El dengue es causado por cuatro serotipos del virus del dengue (DENV-1, DENV-2, DENV-3 y DENV-4), del género Flavivirus. Se transmite principalmente por la picadura de mosquitos *Aedes*, que se reproducen en agua estancada y son más activos durante el día ³. Esta enfermedad constituye un importante problema de salud pública global, con una carga estimada de más de 390 millones de infecciones anuales, según modelos epidemiológicos recientes ^4^. La infección presenta un espectro clínico que varía desde formas asintomáticas hasta cuadros graves con desenlace fatal ^3^^,^^5^. La Organización Mundial de la Salud clasifica la enfermedad en: dengue sin signos de alarma (fiebre, cefalea, dolor retroocular, mialgias, artralgias, náuseas, vómitos y exantema), dengue con signos de alarma (dolor abdominal intenso, vómitos persistentes, acumulación de líquidos, sangrado, letargia o hepatomegalia) y dengue grave, caracterizado por extravasación de plasma, hemorragias severas o daño orgánico significativo ^5^^.^^6^.

Aunque el dengue se asocia principalmente con fiebre y manifestaciones hemorrágicas, puede comprometer el sistema cardiovascular, provocando miocarditis, arritmias, pericarditis y disfunción ventricular, con una incidencia variable de 0,3% a 71% ⁷.

La miocarditis es una complicación poco frecuente pero potencialmente grave, que se manifiesta con disnea, fatiga y dolor torácico. Su diagnóstico se basa en hallazgos clínicos, electrocardiograma, ecocardiografía y biomarcadores cardíacos como troponina ^8^. También se han reportado arritmias, incluyendo bradicardia sinusal, taquicardia supraventricular y bloqueos auriculoventriculares, las cuales suelen ser transitorias ^9^. Además, la disfunción ventricular puede manifestarse con una reducción en la fracción de eyección, detectada mediante ecocardiografía ^10^.

Los mecanismos fisiopatológicos propuestos incluyen invasión viral directa del miocardio, respuesta inmune exacerbada y alteraciones hemodinámicas secundarias a shock hipovolémico ^11^. El tratamiento es de soporte, con monitoreo hemodinámico y manejo cuidadoso de líquidos. En casos severos, puede requerirse tratamiento específico para arritmias o soporte mecánico. La mayoría de las complicaciones cardiovasculares son reversibles tras la resolución de la infección ^12^.

Nuestro reporte tiene el objetivo de presentar un caso de miocarditis asociada a dengue en un paciente joven en una región endémica, describiendo su presentación clínica, evolución y desenlace, con el propósito de resaltar la necesidad de un diagnóstico precoz y manejo oportuno de las complicaciones cardiovasculares en el contexto del dengue.

## CASO CLÍNICO

### Información del paciente

Varón de 21 años, natural y procedente de Iquitos (Perú), sin antecedentes patológicos, quirúrgicos ni consumo de alcohol, tabaco o drogas. No refería viajes recientes ni exposición ocupacional relevante. Consulta por cuadro súbito de un día de evolución caracterizado por fiebre, escalofríos, cefalea, artralgias, náuseas y dolor abdominal persistente en epigastrio. Al ingreso, se observó mal estado general, con facies tóxica, letargia y extremidades frías (figura 1).

**Figura 1:** Línea de tiempo de la evolución clínica del caso

### Hallazgos clínicos

Los signos vitales iniciales mostraron: presión arterial (PA) 70/30 mmHg, presion arterial media (PAM) 43,3 mmHg, frecuencia cardíaca (FC) 84/min, frecuencia respiratoria (FR) 22/min, temperatura 36,5 °C y saturación de oxígeno (SatO₂) de 99%. El examen físico evidenció piel pálida y fría en extremidades distales, así como dolor a la palpación profunda en epigastrio, sin signos de irritación peritoneal. Los exámenes de laboratorio iniciales reportaron: plaquetas: 31 × 10⁹/L; leucocitos: 19,72 × 10⁹/L con linfopenia (7%); AST: 608 U/L; ALT: 381 U/L. La serología rápida mostró IgM negativa, IgG positiva y antígeno NS1 negativo para dengue. El electrocardiograma evidenció ritmo sinusal (FC: 78/min), depresión del segmento PR en DI y DII, junto con supradesnivel del ST difuso (0,5 mm en derivaciones de miembros y >1 mm en precordiales). El QRS midió 80 ms, el eje fue de aproximadamente +60°, y el QTc fue de 365 ms (figura 2). La radiografía de tórax mostró cardiomegalia (índice cardiotorácico: 0,56) y redistribución vascular pulmonar.

**Figura 2:** Electrocardiograma: ritmo sinusal y frecuencia cardíaca: 78 latidos/minuto. Onda P: duración 80 ms (0,08 s) y altura 0.1 mV (1 mm). PR: 160 ms (0,16 s). QRS: 80 ms (0,08 s). Eje cardíaco: isoléctrica en AvL: +60°. QT corregido: 365 ms (0,36). Entre los hallazgos anormales se encontró depresión del segmento PR predominante en DI y DII, supradesnivel del ST difuso de 0,5 mm en derivadas de miembros, y mayor a 1 mm en derivadas precordiales.

### Evaluación diagnóstica

Ante la presencia de hipotensión, dolor abdominal y trombocitopenia severa, se consideró inicialmente dengue con signos de alarma. Los hallazgos electrocardiográficos (depresión del segmento PR y elevación difusa del ST), junto con la disfunción sistólica observada posteriormente, fueron compatibles con una probable miocarditis aguda.

Durante la estancia en la unidad de cuidados intensivos (UCI), se realizó un ecocardiograma que evidenció hipocinesia global difusa y fracción de eyección del ventrículo izquierdo (FEVI) de 35-40%. Los biomarcadores cardíacos mostraron CPK-MB de 72,3 ng/mL (elevada). La reacción en cadena de la polimerasa (PCR) para dengue resultó positiva para DENV-2 al día siguiente, confirmando la etiología viral. No se dispuso de biopsia endomiocárdica.

### Intervención terapéutica

A su ingreso se inició hidratación endovenosa según el protocolo para dengue con signos de alarma, con mejoría tensional transitoria. Dos horas después, la presión arterial descendió a 80/50 mmHg, por lo que fue trasladado a la UCI.

En la UCI se instauró soporte con vasopresores e inotrópicos debido a persistencia de la hipotensión. Posteriormente, el paciente desarrolló dificultad respiratoria severa, requiriendo ventilación mecánica. Se instauró manejo de soporte avanzado para shock mixto (distributivo y cardiogénico) asociado a miocarditis.

### Seguimiento y desenlace

A pesar del soporte vasoactivo en dosis máximas, evolucionó a anuria y shock refractario. El deterioro hemodinámico progresó rápidamente y el paciente falleció pocas horas después de haberse establecido el diagnóstico de miocarditis asociada a infección por DENV-2.

### Perspectiva del paciente

No fue posible recoger la perspectiva del paciente debido a su desenlace fatal; sin embargo, se respeta la confidencialidad y se justifica la publicación por su valor científico y educativo.

## DISCUSIÓN

El dengue, una enfermedad viral transmitida por el mosquito *Aedes aegypti*, afecta principalmente a las regiones tropicales y subtropicales. Aunque la mayoría de los casos son leves, algunos pueden desarrollar complicaciones graves, como alteraciones cardiovasculares, que son poco frecuentes pero preocupantes ^12^. La miocarditis, una complicación fulminante del dengue grave, puede comprometer tanto la estructura como la función del corazón, ocasionando insuficiencia cardíaca o arritmias. Este fenómeno se asocia con mayor morbilidad, especialmente en pacientes previamente infectados por otros serotipos del virus ^12^.

Las manifestaciones cardíacas asociadas al dengue constituyen una complicación poco frecuente; sin embargo, deben ser consideradas, ya que la fuga plasmática puede comprometer gravemente el sistema cardiovascular y ocasionar desenlaces fatales ^(^¹³^)^. En el presente caso, los hallazgos de hipocinesia global y disfunción ventricular sugieren daño miocárdico, lo cual coincide con reportes previos. Un caso similar descrito en un paciente asiático de 49 años presentó miocarditis aguda fulminante con compromiso inicial del ventrículo derecho y progresión al ventrículo izquierdo, que culminó en shock cardiogénico y muerte ^13^. Además, un estudio epidemiológico en Cuba identificó alteraciones electrocardiográficas en un porcentaje importante de pacientes con dengue, destacándose los trastornos del ritmo en el 16,9% de los casos estudiados ^14^^,^^15^.

Como se indica, el dengue puede provocar deterioro funcional del miocardio, tanto en la fase aguda como en formas más graves de la enfermedad. En este paciente, la identificación de hipocinesia global difusa y una fracción de eyección reducida del ventrículo izquierdo a través del ecocardiograma sugiere la posible presencia de miocarditis. Esto concuerda con la literatura, que documenta que la disfunción del ventrículo izquierdo (VI) puede ser causada por miocarditis subclínica, edema miocárdico o mecanismos circulatorios que afectan la contractilidad miocárdica ^11^.

Los casos muestran manifestaciones cardiovasculares raras en el dengue grave, como insuficiencia cardíaca y arritmias. Los hallazgos de hipocinesia y las alteraciones electrocardiográficas coinciden con lo descrito en la literatura, donde se subraya que estas complicaciones suelen estar subestimadas y requieren una vigilancia clínica estrecha. En particular, revisiones regionales han destacado la importancia de un monitoreo adecuado en pacientes con dengue grave debido al riesgo de progresión hacia disfunción cardíaca significativa ^16^.

Es importante mencionar que el paciente presentó un desenlace desfavorable y compartía anomalías electrocardiográficas con otro caso reportado en un varón joven en Argentina, quien desarrolló miocarditis fulminante por dengue, con un patrón ecocardiográfico compatible con Brugada tipo 1 y desenlace fatal. Este hallazgo refuerza la evidencia de que los varones podrían ser más susceptibles a desarrollar complicaciones durante el curso de la enfermedad, en comparación con las mujeres ^17^.

### Implicancias para la salud pública

En Loreto, hasta la Semana Epidemiológica 10 del año 2025 se reportaron más de 4100 casos de dengue, con predominio de formas sin signos de alarma ^18^, pero con un aumento sostenido de casos con signos de alarma y dengue grave, en un contexto clasificado como zona de epidemia. Esta situación evidencia la necesidad de fortalecer la vigilancia clínica y la identificación temprana de signos de alarma, ya que su reconocimiento oportuno permite prevenir complicaciones graves como la miocarditis y reducir la mortalidad asociada ^19^. El caso presentado demuestra cómo la falta de detección precoz de compromiso cardíaco puede influir en desenlaces fatales, lo que subraya la urgencia de optimizar el manejo clínico del dengue en los hospitales de la Amazonía peruana.

Entre las principales limitaciones de este reporte se encuentra la falta de confirmación tisular, debido a la imposibilidad de realizar una autopsia. La ausencia de un estudio posmortem impidió confirmar el diagnóstico histopatológico y descartar diagnósticos diferenciales. Asimismo, no fue posible incluir la perspectiva del paciente, hecho ya consignado previamente en el manuscrito, debido a su desenlace fatal.

En conclusión, se resalta la necesidad de incluir electrocardiogramas y ecocardiografías en la evaluación clínica de pacientes con dengue, ya que la detección temprana de alteraciones cardíacas puede mejorar el pronóstico. Dado que esta enfermedad es endémica en zonas tropicales, resulta crucial que los profesionales de la salud monitoreen activamente las complicaciones cardiovasculares y profundicen en su investigación, con el objetivo de abordar de manera integral tanto la infección viral como sus manifestaciones extrapulmonares, en particular las cardíacas, para reducir la morbilidad y mortalidad asociadas.
